# Editorial: Ion channels and transporters in epilepsy: From genes and mechanisms to disease-targeted therapies

**DOI:** 10.3389/fnmol.2022.1011843

**Published:** 2022-09-21

**Authors:** Hailan He, Tobias Stauber, Weiping Liao, Yuwu Jiang, Yongguo Yu, Jing Peng

**Affiliations:** ^1^Department of Pediatrics, Xiangya Hospital, Central South University, Changsha, China; ^2^Clinical Research Center for Children Neurodevelopmental Disabilities of Hunan Province, Changsha, China; ^3^Department of Human Medicine and Institute for Molecular Medicine, MSH Medical School Hamburg, Hamburg, Germany; ^4^Department of Neurology, Second Affiliated Hospital of Guangzhou Medical University, Guangzhou, China; ^5^Department of Pediatrics, Peking University First Hospital, Beijing, China; ^6^Xinhua Hospital, School of Medicine, Shanghai Jiao Tong University, Shanghai, China

**Keywords:** ion channels, ion transporters, gene, neuronal excitability, epilepsy

Epilepsy is a common, serious neurological disease with affecting more than 50 million people in the world (Singh and Sander, 2020). Neuronal excitability is determined by the flux of ions through ion channels and transporters, and dysfunction of ion homeostasis has been implicated in human epilepsy (Graves, 2006; Wei et al., 2017; Oyrer et al., 2018). The complex pathogenesis of epilepsy is caused by the imbalance of excitation and inhibition of the central nervous system, which is closely related to ion channel and transporters abnormalities (Mizielinska, 2007). Therefore, understanding the role of ion channels and transporter in epilepsy might not only contribute to clarify the mechanism of epileptogenesis but also provide potential targets for the precise treatment of epilepsy. Within this context, we launched our Research Topic on April 23th, 2021, and invited researchers to address *Ion channels and transporters in epilepsy: From genes and mechanisms to disease-targeted therapies*. Despite all the hardships, and uncertainty caused by the COVID-19 pandemic, we have received diverse and insightful manuscripts. In a cohort of 221 pediatric epilepsy, Jiang et al. described that genetic testing may help identify the molecular etiology of early onset epilepsy and developmental delay/intellectual disability and further aid to choose the appropriate treatment strategy for patients. Liu X.-R. et al. studied the role of *GRIN2A* gene in idiopathic generalized epilepsies and the potential underlying mechanism for phenotypic variation. Their results suggested a relationship between the severity of gain-of-function effects of GluN2A and the severity of the phenotypes as well as a link between the location of the variations in the different domains of the GluN2A protein and the epileptic phenotypes. Kessi et al. reviewed the contribution of hyperpolarization-activated cyclic nucleotide-gated (HCN) channelopathies in different epileptic syndromes. They update knowledge about the human genetic changes, genotype–phenotype correlations, the available animal models, and the drugs available for each subtype of channel in the HCN family. Xie et al. identified five HCN1 channel variants in five patients with epilepsy, four of which were novel. They further demonstrated that these mutations affect the biophysical properties of HCN1 channels and neuronal excitability *in vitro* experiments. Yang M. et al. showed an increased expression of Beclin1 upon epileptic activity in human and mouse brain. The authors also showed haploinsufficiency of *Beclin1* results in decreased seizure susceptibility accompanied by decreased frequency and amplitude in miniature excitatory synaptic currents and decreased number of dendritic spines in hippocampal neurons in *Beclin*1^+/−^ mice. Xiao et al. reported 30 unrelated patients with novel variants in the *KCNQ2* gene, including 19 single nucleotide variations and 11 copy number variants who experienced seizures or neurological development delay. Their study expands the genotypic and phenotypic spectrum of *KCNQ2*-related disorder. Yang et al. described the phenotypes of *GABRG2*-related epilepsy were ranged from mild febrile seizures to severe epileptic encephalopathies. Seizure outcome was favorable in most patients with *GABRG2* variants, and most patients benefited from treatment with valproate and/or levetiracetam. Zeng et al. analyzed the phenotypic spectrum, treatment and prognosis of 72 Chinese epilepsy children with *SCN2A* variants. The epilepsy phenotypes of these patients with *SCN2A* variants were variable, ranging from febrile seizures (plus), benign epilepsy to epileptic encephalopathy, and 79.1% patients had developmental delay. Of the patients with *SCN2A* variants, 22.6% had achieved seizure control with valproate, 28.9% with oxcarbazepine, while 6 patients had seizure worsening by oxcarbazepine. Weng et al. presented recent evidence of iPSC analysis of certain ion channel mutations found in humans with epilepsy and genes related to mTOR signaling. Recent years, there has been a great increase in the use of human iPSC models of genetic epilepsy syndromes, so this review is a timely contribution. Chen et al. described detailed genotype-phenotype information for a group of 41 patients with pathogenic *SCN1A* variants. The authors classified these 41 epilepsy patients into two groups, Dravet syndrome and non-Dravet syndrome, and retrospectively compared the phenotypic differences between the two groups. Ma et al. reported 22 epileptic patients with *SCN1A* variants, including 12 novel variants. Interestingly, two patients in their cohort displayed rare phenotypes, benign epilepsy with centrotemporal spikes and atypical childhood epilepsy with centrotemporal spikes. Therefore, studies of both Chen et al. and Ma et al. expand the genotypes and phenotypes of *SCN1A*-related epilepsy. Almog et al. studied the electrophysiological characterization of inhibitory and pyramidal neurons in CA1 hippocampal region of *Scn1a*A^1783V/+^ knock-in mouse model of Dravet syndrome. Their data reveal synaptic and excitability alterations in both CA1 excitatory neurons and CA1 stratum-oriens interneurons in *Scn*1*a*^A1783V/+^ mice, supporting global homeostatic changes within the CA1 microcircuit that may partially compensate for the Dravet syndrome-related interneurons hypoexcitability. Guzman et al. studied the functional characterization of *CLCN4* variants associated with X-linked intellectual disability and epilepsy. Their results showed these mutations led to a variety of changes in ClC-4 function, ranging from gain/loss of function and impaired heterodimerization with ClC-3 to subtle impairments in transport functions. These findings provide insights into understanding the linkage between *CLCN4* mutations and their associated neurological phenotypes. Liu X. et al. investigated the mechanism that underlie a protective role of neural precursor cell expressed developmentally down-regulated gene 4-like (NEDD4-2) against the endoplasmic reticulum (ER) stress and seizure susceptibility with the RER1 as a possible mediator. The authors found that Nedd4-2 haploinsufficiency in mice impairs the ubiquitination of Rer1 and increases the susceptibility to ER stress and seizures. Their study enriches our knowledge on the underlying mechanism of seizure contributing to ER stress.

Taken together, a total of fourteen articles were published under this collection, which have improved our understanding about the molecular basis underlying genetic epilepsy caused by mutations in ion channels and transporters, as well as related clinical problems. We hope that all the selected clinical and basic studies published in this Research Topic have provided insights on the link between ion channels, ion transporters and epilepsy, with promising outcomes and future perspectives. However, deciphering the mechanisms and understanding the role of the ion channels and transporters in epilepsy is a long way to go.

## Author contributions

All authors listed have made a substantial, direct, and intellectual contribution to the work and approved it for publication.

## Funding

This study was supported by the National Natural Science Foundation of China (Nos. 81771409 and 82071462).

## Conflict of interest

The authors declare that the research was conducted in the absence of any commercial or financial relationships that could be construed as a potential conflict of interest.

## Publisher's note

All claims expressed in this article are solely those of the authors and do not necessarily represent those of their affiliated organizations, or those of the publisher, the editors and the reviewers. Any product that may be evaluated in this article, or claim that may be made by its manufacturer, is not guaranteed or endorsed by the publisher.
